# Evaluation of the Novel Synthetic Tyrosinase Inhibitor (*Z*)-3-(3-bromo-4-hydroxybenzylidene)thiochroman-4-one (MHY1498) *In Vitro* and *In Silico*

**DOI:** 10.3390/molecules23123307

**Published:** 2018-12-13

**Authors:** EunJin Bang, Sang-Gyun Noh, Sugyeong Ha, Hee Jin Jung, Dae Hyun Kim, A Kyoung Lee, Min Kyung Hyun, Dongwan Kang, Sanggwon Lee, Chaeun Park, Hyung Ryong Moon, Hae Young Chung

**Affiliations:** College of Pharmacy, Pusan National University, Busan 46241, Korea; ejbang@pusan.ac.kr (E.B.); rskrsk92@naver.com (S.-G.N.); tnrn34@hanmail.net (S.H.); hjjung2046@pusan.ac.kr (H.J.J.); bioimmune@hanmail.net (D.H.K.); lak000@naver.com (A.K.L.); Mminn94@naver.com (M.K.H.); 3607@pusan.ac.kr (D.K.); lsk3232@pusan.ac.kr (S.L.); sauryyyy@pusan.ac.kr (C.P.); mhr108@pusan.ac.kr (H.R.M.)

**Keywords:** (*Z*)-3-(3-bromo-4-hydroxybenzylidene)thiochroman-4-one, tyrosinase, *in silico* docking simulation, B16F10, α-melanocyte-stimulating hormone

## Abstract

Tyrosinase is a key enzyme in melanin synthesis, catalyzing the initial rate-limiting steps of melanin synthesis. Abnormal and excessive melanin synthesis is the primary cause of serious skin disorders including melasma, senile lentigo, freckles, and age spots. In attempts to find potent and safe tyrosinase inhibitors, we designed and synthesized a novel compound, (*Z*)-3-(3-bromo-4-hydroxybenzylidene)thiochroman-4-one (MHY1498), and evaluated its tyrosinase inhibitory activity *in vitro* and *in silico*. The chemical structures of (*Z*)-3-benzylidenethiochroman-4-one analogues, including the novel compound MHY1498, were rationally designed and synthesized as hybrid structures of reported potent tyrosinase inhibitors, which were confirmed both *in vitro* and *in vivo*: (*Z*)-5-(substituted benzylidene)thiazolidine-2,4-diones (Compound A) and 2-(substituted phenyl)benzo[*d*]thiazoles (Compound B). During screening, MHY1498 showed a strong dose-dependent inhibitory effect on mushroom tyrosinase. The IC_50_ value of MHY1498 (4.1 ± 0.6 μM) was significantly lower than that of the positive control, kojic acid (22.0 ± 4.7 μM). *In silico* molecular multi-docking simulation and inhibition mechanism studies indicated that MHY1498 interacts competitively with the tyrosinase enzyme, with greater affinity for the active site of tyrosinase than the positive control. Furthermore, in B16F10 melanoma cells treated with α-melanocyte-stimulating hormone, MHY1498 suppressed both melanin production and tyrosinase activity. In conclusion, our data demonstrate that MHY1498, a synthesized novel compound, effectively inhibits tyrosinase activity and has potential for treating hyperpigmentation and related disorders.

## 1. Introduction

Melanogenesis is the process that leads to the production of the dark macromolecular pigment melanin by melanocytes. Melanin synthesis occurs via a serial process of enzymatic catalyses and chemical reactions [1]. The melanogenesis process is initiated by the activity of the enzyme tyrosinase, which catalyzes the oxidation of tyrosine to dopaquinone, a melanin precursor [2]. Melanin determines skin pigmentation and normally functions to prevent skin injury through the absorption of harmful UV radiation. The photochemical properties of melanin make it an excellent photoprotectant, as it absorbs harmful UV rays and emits this energy as harmless heat through a process referred to as ‘ultrafast internal conversion’ [3]. However, abnormal and excessive accumulation of melanin may result in skin disorders such as hyperpigmentation, melasma, freckles, age spots, and senile lentigo [1,4,5,6]. Therefore, regulation of melanogenesis is an important strategy to consider in the treatment of aesthetic and serious skin disorders associated with abnormal skin pigmentation.

Tyrosinase is a copper-containing enzyme found widely in nature. It is a rate-limiting enzyme that catalyzes the two initial sequential oxidations of l-tyrosine in melanin biosynthesis [7]. During melanogenesis, tyrosinase interacts primarily with l-tyrosine as its substrate and catalyzes the hydroxylation of l-tyrosine to 3,4-dihydroxy-l-phenylalanine (l-DOPA) and the oxidation of l-DOPA to generate DOPA quinine [8,9,10]. Due to its rate-limiting role in melanogenesis, efforts have been made to develop tyrosinase inhibitors for cosmetic and therapeutic purposes, and in recent decades several natural and synthetic tyrosinase inhibitors have been identified [7]. These include tyrosinase inhibitors like hydroquinone, ascorbic acid derivatives, azeleic acid, retinoids, arbutin, kojic acid, resveratrol, and polyphenolic compounds [11,12,13,14]. However, some widely known tyrosinase inhibitors, such as whitening hydroquinone, kojic acid, and arbutin, have been reported to elicit undesirable side effects, including dermatitis, cytotoxicity, and the development of cancers [7,15,16]. Therefore, it is important that safe and effective tyrosinase pharmacological inhibitors are identified and characterized.

In our previous studies, our lab synthesized (*Z*)-5-(substituted benzylidene)thiazolidine-2,4-diones (compound A) and 2-(substituted phenyl)benzo[*d*]thiazoles (compound B) as potential tyrosinase inhibitors and demonstrated their potent inhibitory activity both *in vitro* and *in vivo* [17,18,19,20]. Compound A has a characteristic α-thio-β-(hydroxyl-substituted phenyl)-α,β-unsaturated carbonyl structure, and compound B has a 2-(hydroxyl-substituted phenyl)benzo[*d*]thiazolyl moiety. Present in compound A, *N*-phenylthiourea was reported to inhibit a catechol oxidase enzyme belonging to the tyrosinase type-3 copper protein group via its sulfur atom binding to both copper ions located in the tyrosinase active site [21]. This led to the synthesis of a hydroxyl- and an alkoxy-substituted phenyl-benzol[*d*]thiazole compound, in which the sulfur atom of the benzothiazole ring can act as a chelator of copper ions in the active site of tyrosinase, similar to *N*-phenylthiourea. Additionally, because the nitrogen atom of the substituted phenyl-benzo[*d*]thiazole derivatives can form a quaternary ammonium ion at physiological pH, similar to tyrosine and l-DOPA, the nitrogen atom might act as a positive center capable of interacting with anionic or partially anionic groups of amino acid residues present in the tyrosinase active site. Therefore, hydroxyl- and alkoxy-substituted phenyl-benzo[*d*]thiazole derivatives might serve as potent competitive tyrosinase inhibitors. The imidazole ring in compound B was reported to possess significant inhibitory activity against tyrosine [20]. Consequently, we synthesized the compound (*Z*)-5-(2,4-dihydroxyvenzylidene)thiazolidine-2,4-dione, substituting NH for S in the imidazole ring [20]. Based on structure–activity predictions, according to which these characteristic structures of compounds A and B would promote strong tyrosinase inhibition, hybrid structures of these two compounds were designed, such as (*Z*)-3-(hydroxyl-substituted benzylidene) thiochroman-4-one analogues, to find novel tyrosinase inhibitors.

The purpose of this study was to screen, identify, and characterize synthesized potent (*Z*)-3-(hydroxyl-substituted benzylidene)thiochroma-4-one analogue tyrosinase inhibitors. In screening, we found a novel compound, MHY1498, that demonstrated strong tyrosinase inhibition activity in a cell-free system and we evaluated the molecular interaction between tyrosinase and MHY1498 by *in silico* molecular multi-docking experiments. We found that MHY1498 interacts with the catalytically active site of tyrosinase with greater affinity than the positive control compound kojic acid. Tyrosinase inhibitory activity was also evaluated in B16F10 murine melanoma cells, showing that MHY1498 was effective at preventing α-melanocyte-stimulating hormone (α-MSH)-induced melanogenesis. In conclusion, the data indicate that MHY1498 may be a strong tyrosinase inhibitor with potential for use in the treatment of hyperpigmentation disorders.

## 2. Results

**Figure 1:**
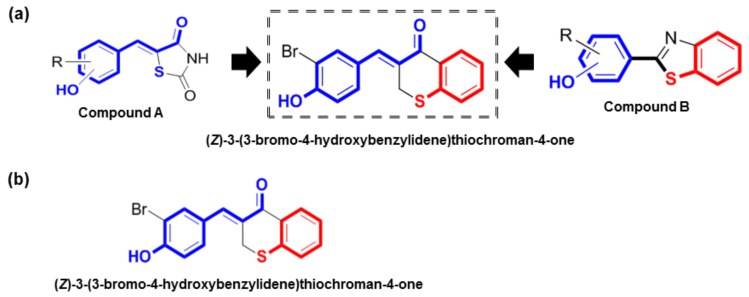
Rationale for the design and synthesis of (*Z*)-3-(3-bromo-4-hydroxybenzylidene)thiochroman-4-one (MHY1498). (**a**) Rationale for the design of MHY1498; (**b**) chemical structure of (*Z*)-3-(3-bromo-4-hydroxybenzylidene)thiochroman-4-one.

In previous studies, we synthesized (*Z*)-5-(substituted benzylidene)thiazolidine-2,4-diones (compound A) and 2-(substituted phenyl)benzo[*d*]thiazoles (compound B) as potential tyrosinase inhibitors (Figure 1a) [17,18,19,20]. Our *in vitro* and *in vivo* studies demonstrated that these compounds had potent tyrosinase inhibitory effects [17,18,19,20]. Compound A has a characteristic α-thio-β-(hydroxyl-substituted phenyl)-α,β-unsaturated carbonyl structure, and its *N*-phenylthiourea moiety has been reported to inhibit a catechol oxidase enzyme belonging to the group of tyrosinases that contain a copper group in the tyrosinase active site. Compound B has a 2-(hydroxyl-substituted phenyl)benzo[*d*]thiazolyl moiety, and its imidazole ring has been reported to exhibit notable inhibitory activity against tyrosinase. Based on the rationale that these characteristic structures may promote strong tyrosinase inhibition, hybrid structures of these two (*Z*)-3-(hydroxyl-substituted benzylidene)thiochroman-4-one analogs were rationally designed and synthesized to identify potent tyrosinase inhibitors (Figure 1a).

**Figure 2:**
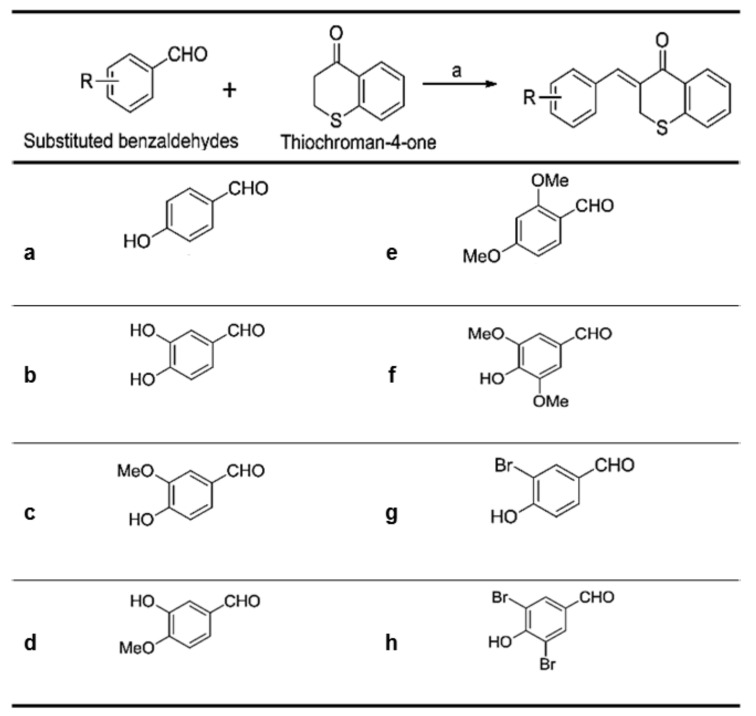
Substitution pattern of the (*Z*)-3-(hydroxyl-substituted benzylidene)thiochroman-4-one analogs.

**Figure 3:**
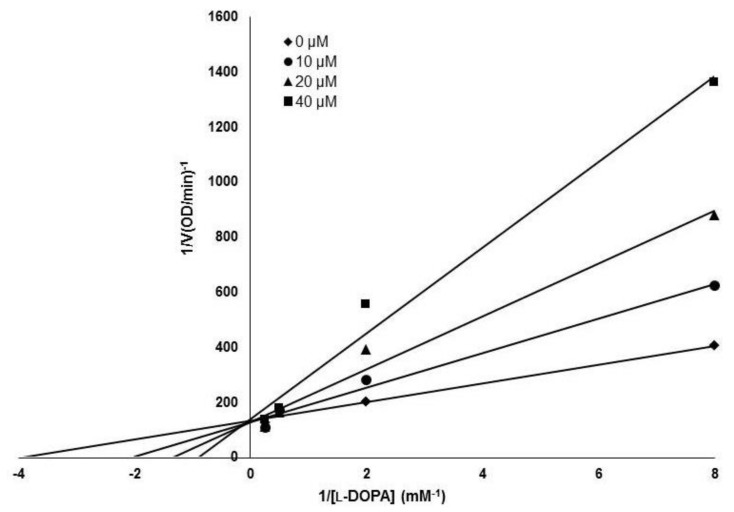
Lineweaver–Burk plot for the tyrosinase inhibitory mechanism of MHY1498. Tyrosinase inhibition was examined at different concentrations (0, 10, 20 and 40 μM); l-DOPA was used as a substrate for the experiment.

As shown in Figure 2, (*Z*)-3-(hydroxyl-substituted benzylidene)thiochroman-4-one analogue compounds (Figure 2**)**, were synthesized via a Claisen–Schmidt condensation reaction. Appropriate hydroxyl-substituted benzaldehydes were treated with thiochroman-4-one under acidic conditions (1.0 M HCl solution in acetic acid) with or without the addition of 12.0 M HCl aqueous solution to generate the desired compounds (Figure 2), with yields of 14.1% to 38.7%. Products with a (*Z*)-configuration were predominantly generated, likely because of steric hindrance. For the synthesis of the novel compound g (Figure 2), or MHY1498 ((*Z*)-3-(3-bromo-4-hydroxybenzylidene)thiochroman-4-one), a solution of 3-bromo-4-hydroxybenzaldehyde and thiochroman-4-one in 1.0 M HCl in acetic acid (0.5 mL) and 12.0 M hydrochloric acid (0.02 mL) was heated at 60 °C for three days. The reaction mixture was partitioned between dichloromethane and water, and the organic layer was dried over anhydrous MgSO_4_, filtered, and evaporated in vacuo. The resultant residue was recrystallized in methanol and water to give MHY1498 as a yellow solid. The chemical structure of the novel compound MHY1498 is shown in Figure 1b.

### 2.1. Inhibitory Effect of MHY1498 on Mushroom Tyrosinase

The inhibitory activity of (*Z*)-3-(hydroxyl-substituted benzylidene)thiochroman-4-one analogues on mushroom tyrosinase was evaluated using kojic acid as a positive control compound. As tyrosinase is an enzyme that mediates the biochemical conversion of both l-tyrosine and l-DOPA to dopachrome in melanogenesis, tyrosinase activity was evaluated by measuring the absorbance of the generated dopachrome. The inhibitory activity of the synthesized analogue compounds on mushroom tyrosinase was screened in a cell-free system. The results indicated that MHY1498 (compound g) had stronger inhibitory activity against tyrosinase than kojic acid (Table 1), with values of 90.7 ± 4.1% inhibition for MHY1498 and 77.5 ± 17.4% for kojic acid. In addition, MHY1498 dose-dependently suppressed tyrosinase activity in a concentration range of 0–20 μM when the activity was measured using l-DOPA as the tyrosinase substrate (data not shown). Compared to kojic acid (10 μM), MHY1498 showed significantly decreased tyrosinase activity (data not shown). The IC_50_ values for MHY1498 and kojic acid were calculated as shown in Table 2. The IC_50_ was 4.1 ± 0.6 μM for MHY1498, and 22.0 ± 4.7 μM for kojic acid, indicating that MHY1498 is approximately a five-fold more potent tyrosinase inhibitor than kojic acid. To investigate the inhibitory mechanism of MHY1498, Lineweaver–Burk double-reciprocal plots were evaluated using different concentrations of l-tyrosine substrate (0.25, 0.5, 1, 2, 4, 8, and 16 mM). The change in absorbance was measured in a time-dependent manner, and the results were plotted double-reciprocally as 1/V versus 1/[S] (Figure 3). The plot produced four different lines at different concentrations of MHY1498 (0, 10, 20, and 40 μM), with different slopes intersecting on the same vertical axis, indicating that MHY1498 inhibited tyrosinase binding in a competitive manner. These data suggest that MHY1498 strongly inhibits tyrosinase activity in a dose-dependent and competitive manner.

Tyrosinase activity was measured using l-DOPA as the substrate. * The individual IC_50_ values were calculated as the concentration at which tyrosinase activity was inhibited by 50%. Values shown are the mean ± S.E. of three determinations.

### 2.2. In Silico Molecular Docking Simulation of MHY1498 on Tyrosinase

**Figure 4:**
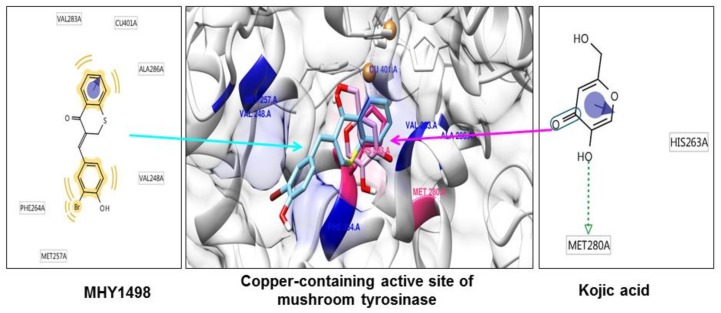
*In silico* docking simulation between MHY1498 or kojic acid and tyrosinase. The computational structure prediction for mushroom tyrosinase is shown in the middle, with MHY1498 bound close to the copper-containing tyrosinase active site. The two brown spheres indicate copper ions at the active site. Cyan denotes MHY1498 binding sites, and red indicates kojic acid binding sites. The binding residues of MHY1498 (left panel) and kojic acid (right panel) were analyzed using Autodock Vina, AutoDock 4, and Dock 6.

As tyrosinase is an essential enzyme for melanogenesis, we used *in silico* multi-docking simulation programs to investigate whether MHY1498 can bind directly to tyrosinase and inhibit its activity with greater affinity. The computation docking simulation results for tyrosinase and binding compounds (MHY1498 and kojic acid) are shown in Figure 4. The computational structure prediction of mushroom tyrosinase is shown in the middle panel, where two brown spheres indicate copper ions at the active site. MHY1498 (cyan) appeared to closely interacts with the copper-containing active site predicted by Autodock Vina, AutoDock 4, and Dock 6 indicated that it had a greater inhibitory potency and binding affinity than the control. Possible residues involved in hydrophobic interactions between MHY1498 and tyrosinase include VAL283A, CU401A, ALA286A, MET257A, PHE264A, and VAL248A, and the critical interactive residues that form hydrogen bonds between kojic acid and tyrosinase are HIS263A and MET280A. These residues may have key functions and major effects on the binding affinity. Although more studies are required to understand the mechanism underlying MHY1498 inhibition of tyrosinase activity, the molecular docking simulation results suggest that MHY1498 binds directly to the copper active site by forming hydrophobic bonds. The greater binding affinity indicated by the lower docking score of MHY1498 explains the stronger inhibitory activity of MHY1498 against tyrosinase compared to kojic acid.

The multi-docking scores were generated using three different simulation programs, i.e., Autodock Vina, Autodock 4, and Dock 6. The binding energies predicted by these three programs between MHY1498 and tyrosinase were −6.6, −7.0, and −30.5 kcal/mol, respectively, and −5.6, −5.2, −27.6 kcal/mol, respectively, for kojic acid (Table 3). The results are consistent with the cell-free *in vitro* data demonstrating a greater percent inhibition of MHY1498 compared to kojic acid. Although the MHY1498 inhibitory mechanism has not been clearly elucidated by X-crystallography, computational docking simulation has been established as a powerful tool to screen and evaluate new pharmacological agents. Docking simulation results provide not only the biding sites, but also the binding energies generated between a chemical compound and an enzyme, which is informative prior to the initiation of *in vitro* cell-based experiments. Although the predictions are not always accurate and require biological experimental verification, this method reduces time and effort when screening for effective compounds. Consequently, our data, providing predictive docking energy and revealing binding residues, support the conclusion that MHY1498 exhibits stronger tyrosinase inhibition than kojic acid because of its higher affinity for the tyrosinase active site.

### 2.3. Effects of MHY1498 on Cellular Viability of B16F10 Melanoma Cells

**Figure 5:**
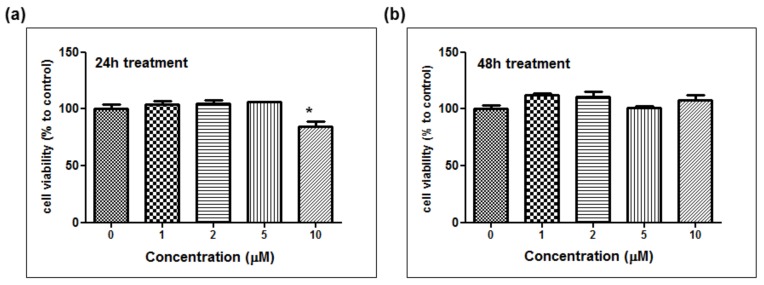
The effect of MHY1498 on the viability of B16F10 melanoma cells. The cells were treated with different concentrations of MHY1498 (0, 1, 2, 5, and 10 μM) for 24 (**a**) and 48 (**b**) h and assessed for cytotoxicity using an MTT assay. The values represent the mean ± SD for 3 experiments (* *p* < 0.05, control vs. MHY1498 (10μM). The data are expressed as percent compared to the control.

The depigmentation activity of MHY1498 was evaluated using B16F10 murine melanoma cells. The cytotoxic effect of MHY1498 was measured at various concentration of MHY1498 (1, 2, 5, and 10 μM) for 24 and 48 h (Figure 5a,b). The results showed that MHY1498 was not cytotoxic in B16F10 cells up to a concentration of 10 μM, allowing us to assess the effects of MHY1498 up to 10 μM concentration for up to 48 h. The seemingly decrease in cell viability at 10 μM after 24 h of treatment appeared to be due to experimental variations.

### 2.4. Effects of MHY1498 on B16F10 Melanoma Cells

**Figure 6:**
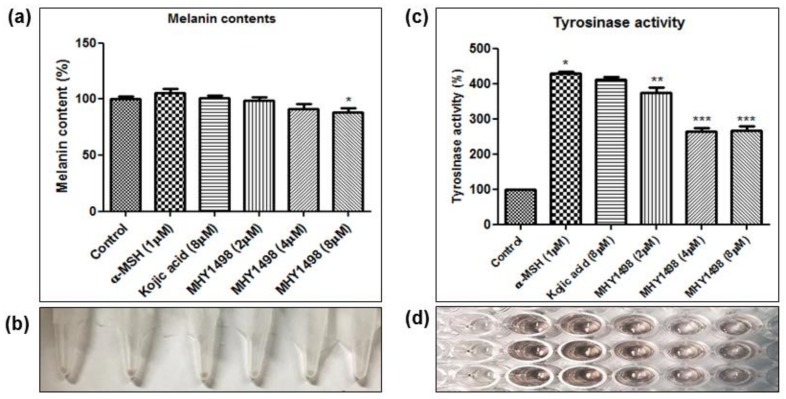
Effects of MHY1498 on melanin synthesis in B16F10 melanoma cells. (**a**) B16F10 cells were incubated with 2, 4 or 8 μM MHY1498 for 48 h. The melanin content in B16F10 cells was determined as described. The melanin content of control cells was defined as 100%. Kojic acid was used as a positive control. All data are shown as mean ± SD (* *p* < 0.001, control vs. α-MSH, ** *p* < 0.05, MHY1498 (2 μM) vs. α-MSH, *** *p* < 0.001, MHY1498 (4, 8 μM) vs. α-MSH). (**b**) Cellular pigmentation in B16F10 cells was notably suppressed in the MHY1498 treatment group (8 μM). (**c**) The cellular tyrosinase activity was measured after treatment with 2, 4, 8 μM MHY1498 for 48 h. All data are shown as mean ± SD (* *p* < 0.05, compared to control cells). (**d**) Tyrosinase activity in B16F10 cells was evaluated by measuring the absorbance of the produced dopachrome at 492 nm.

To evaluate the depigmentation effects of MHY1498, melanin content and tyrosinase activity were assessed in B16F10 cells after treatment with various concentrations of MHY1498 (0, 2, 4 and 8 μM), in the presence or absence of 1 μM α-MSH. The concentration for *in vitro* assessment was selected near the IC_50_ value (4.1 ± 0.6 μM) for MHY1498, below the dose that did not seem to elicit cytotoxicity (10 μM). Treatment with 1 μM α-MSH induced an increase in melanin content in B16F10 melanoma cells, and co-treatment with MHY1498 marginally decreased melanin content in a dose-dependent manner (Figure 6a). Total melanin synthesis resulting from α-MSH treatment was discernable and showed that MHY1498 decreased α-MSH-induced melanin content (Figure 6b). The results indicate that MHY1498, but not kojic acid, effectively and significantly decreased melanin synthesis at a concentration of 8 μM in a cell culture system. Tyrosinase activity increased significantly with 1 μM α-MSH treatment. In contrast to kojic acid, treatment of MHY1498 significantly decreased tyrosinase activity at both 4 and 8 μM concentrations (Figure 6c) and was visually discernible by the suppressed amount of dopachrome produced (Figure 6d). The results suggest that MHY1498 treatment effectively inhibited α-MSH-induced cellular melanogenesis. Collectively, these results demonstrate that MHY1498 may be a potent compound for the modulation of melanogenesis via inhibition of tyrosinase.

## 3. Materials and Methods

### 3.1. Materials

Kojic acid, mushroom tyrosinase, l-tyrosinase, l-DOPA, α-melanocyte-stimulating hormone (α-MSH), and other chemical reagents were purchased from Sigma (St. Louis, MO, USA).

### 3.2. Synthesis of (Z)-3-(3-bromo-4-hydroxybenzylidene)thiochroman-4-one (MHY1498)

For the synthesis of compound g (MHY1498, (*Z*)-3-(3-bromo-4-hydroxybenzylidene)thiochroman-4-one), a solution of 3-bromo-4-hydroxybenzaldehyde (100 mg, 0.50 mmol) and thiochroman-4-one (53 μL, 0.40 mmol) in 1.0 M HCl acetic acid (0.5 mL) and 12.0 M hydrochloric acid (0.02 mL) was heated at 60 °C for three days. The reaction mixture was divided between dichloromethane and water, and the organic layer was dried over anhydrous MgSO_4_, filtered, and evaporated in vacuo. The resultant residue was recrystallized in methanol and water to give MHY1498 (35.9 mg, 26%) as a yellow solid.

### 3.3. In Silico Docking Simulation of Tyrosinase and Inhibitory Compounds

For the *in silico* protein–ligand docking simulation, we used Autodock Vina (AutoDock Vina (Ver 1.1.2, open source website, vina.scripps.edu), Autodock 4 (AutoDock 4.2.6, OpenEye Scientific Software, SantaFe, NM, USA), and Dock 6 (Dock 6, official UCSF website: http://dock.compbio.ucsf.edu) using the crystal structure of *Agaricus bisporus* (PDB ID: 2Y9X) to simulate the 3D structure of tyrosinase. Docking simulations were performed between tyrosinase and either MHY1498 or kojic acid. The compounds were prepared for docking simulation, and charges were calculated. The prediction of possible hydrogen binding residues between tyrosinase and the compounds was generated using pharmacophores.

### 3.4. Measurement of Mushroom Tyrosinase Activity

The inhibitory effect of MHY1498 was examined against mushroom tyrosinase. In the tyrosinase inhibition assay, l-tyrosine or l-DOPA was used as a substrate. As tyrosinase is an enzyme that mediates the conversion of both l-tyrosine and l-DOPA to dopachrome, enzymatic activity was monitored by dopachrome formation at 492 nm in the presence of a potent tyrosinase inhibitor. The methods used were as follows: An aqueous solution of mushroom tyrosinase (200 U, 20 µL) was added to the wells of transparent 96-well plates containing 200 µL of a reaction mixture of 1 mM l-tyrosine, 50 mM phosphate buffer (pH 6.5), and the test material (kojic acid or MHY1498) at different concentrations [10–50 µM]). The mixture was incubated at 37 °C for 30 min, and the dopachrome was determined by measuring the absorbance at 492 nm, using a microplate reader (GENios; Tecan Instruments, Salzburg, Austria). The inhibitory mechanism of MHY1498 was evaluated using a kinetic assay with different concentrations of l-tyrosine and MHY1498.

The IC_50_ was calculated from experiments performed with different MHY1498 doses. A tyrosinase kinetic assay was performed to determine the inhibitory mechanism of MHY1498. Various concentrations of l-DOPA (1, 2, 4 and 8 mM) were used for the inhibition assay. After examination, each value was converted into its reciprocal form following Lineweaver–Burk plots. The results showed the plot of 1/*V* versus 1/[S], and the intersection of each plot was used to determine the inhibitory mechanism.

### 3.5. Cell Culture and Viability

Murine melanoma B16F10 cells were purchased from the American Type Culture Collection (Manassas, VA, USA). The cells were cultured in Dulbecco’s Modified Eagle’s Medium (DMEM) containing 5% fetal bovine serum (FBS, Gibco, NY, NY, USA) in 5% CO_2_ at 37 °C. Cell viability was analyzed using the MTT (3-(4,5-Dimethylthiazol-2-yl)-2,5-Diphenyltetrazolium Bromide) assay. The cells were plated in each well of transparent 96-well plates at a density of 5 × 104 cells/mL and treated with MHY1498 at different concentrations, with the addition of the MTT solution. After a 1 h of incubation, the absorbance was measured at 560 nm using a microplate reader.

### 3.6. Determination of Cellular Melanin Content and Tyrosinase Activity

The intracellular melanin levels were determined using B16F10 cells. B16F10 cells were plated on 35 mm dishes at 5 × 10^4^ cells/mL, incubated with MHY1498 for 2 h, and treated with 1 μM α-melanocyte-stimulating hormone (α-MSH) for 48 h. After washing twice with PBS, the cells were evaluated either for melanin content or tyrosinase activity. For melanin content, the samples were dissolved in 500 µL of 1 M NaOH, incubated at 60 °C for 1 h, and mixed to solubilize melanin. Melanin content in the samples was determined by measurement of the absorbance at 405 nm. For tyrosinase activity, the cells were lysed in 90 μL of 50 mM sodium phosphate buffer (pH 6.8) containing 5 µL of 1% Triton 100X and 5 μL of 0.1 mM phenylmethylsulfonyl fluoride (PMSF) The samples were frozen at −80 °C for 30 min. After thawing and mixing, cellular extracts were obtained by centrifugation at 12,000 × g for 30 min at 4 °C. The supernatants (80 μL) with 20 μL of l-DOPA (2 mg/mL) were transferred to a 96-well plate, and absorbance was measured at 492 nm every 10 min for 1 h at 37 °C.

## 4. Conclusions

In summary, the results of the current study indicate that the novel compound MHY1498 is a potent inhibitor of tyrosinase, a rate-limiting enzyme in melanin biosynthesis. *In vitro* and *in silico* evidence showed that tyrosinase activity was strongly suppressed, and we further recorded notable binding affinity between tyrosinase and MHY1498, suggesting that MHY1498 may be a potent tyrosinase inhibitor with potential for use as a cosmetic and therapeutic agent for hyperpigmentation-related disorders.

## Figures and Tables

**Table 1:** Effects of MHY1498 on mushroom tyrosinase activity. l-tyrosine was used as the substrate for mushroom tyrosinase and was added to the mixture as a test material. The inhibitory activity of mushroom tyrosinase was measured after treatment with the synthesized compounds and kojic acid (50 μM). The values shown are the mean ± SD of three determinations.

Compounds	50μM		
	Mean ± SD (%)		
**a**	81.6	±	1.0
**b**	45.0	±	4.7
**c**	25.8	±	3.6
**d**	91.5	±	6.2
**e**	20.3	±	4.5
**f**	15.6	±	23.4
**g**	90.7	±	4.1
**h**	8.5	±	8.2
**Kojic acid**	77.5	±	17.4

**Table 2:** Inhibitory effects on mushroom tyrosinase activity and related IC_50_ values of kojic acid and MHY1498.

Compounds	Concentration	Inhibition Percentage (%)	IC_50_ (μM) *
**MHY1498**	0.1 μM	6.2 ± 6.3	4.1 ± 0.6
	0.5 μM	15.6 ± 7.0	
	1 μM	21.6 ± 7.4	
	2 μM	28.8 ± 7.4	
	5 μM	54.3 ± 5.1	
**Kojic acid**	1 μM	3.0 ± 5.7	22.0 ± 4.7
	2 μM	6.3 ± 2.3	
	5 μM	13.1 ± 3.7	
	10 μM	21.4 ± 10.1	
	20 μM	44.2 ± 5.8	

* 50% inhibitory concentration (IC_50_).

**Table 3:** Multi-docking scores and the major tyrosinase-interactive residues of MHY1498 and kojic acid.

Compounds	Binding Energy (kcal/mol) (Autodock Vina/AutoDock4/Dock6)	Main Interactive Residues
**MHY1498**	−6.6/−7.0/−30.5	VAL283A, CU401A, ALA286A, MET257A, PHE264A, VAL248A
**Kojic acid**	−5.6/−5.2/−27.6	HIS263A, MET280A
